# Atmospheric pressure microwave (915 MHz) plasma for hydrogen production from steam reforming of ethanol

**DOI:** 10.1038/s41598-024-65874-9

**Published:** 2024-06-28

**Authors:** Robert Miotk, Bartosz Hrycak, Dariusz Czylkowski, Mariusz Jasiński, Mirosław Dors, Jerzy Mizeraczyk

**Affiliations:** 1 Institute of Fluid Flow Machinery, Polish Academy of Sciences, 80-231 Gdańsk, Poland Fiszera 14,; 2 Department of Marine Electronics, Gdynia Maritime University, 81-225 Gdynia, Poland Morska 81-87,

**Keywords:** Hydrogen production, Ethanol, Microwave plasma, 915 MHz, Energy yield, Simulations of the distribution of the electric field, Hydrogen energy, Electrical and electronic engineering

## Abstract

This work presents experimental results on the energy efficiency in hydrogen production using atmospheric microwave plasma (915 MHz) through steam reforming of ethanol. Ethanol was chosen as a liquid hydrogen carrier due to its high hydrogen atom content, low cost, and wide availability. The experimental work began with the maximization of an energy efficiency of the used microwave plasma source. The process of maximization involved determining a position of a movable plunger that ensures the most efficient transfer of microwave energy from a microwave source to the generated plasma in the microwave plasma source. The aim of the investigations was to test the following working conditions of the microwave plasma source: absorbed microwave power *P*_A_ by the generated plasma (up to 5.4 kW), the carrier gas volumetric flow rate (up to 3900 Nl/h), and the amount of the introduced ethanol vapours on the efficiency of hydrogen production (up to 2.4 kg/h). In the range of tested working conditions, the highest energy yield for hydrogen production achieved a rate of 26.9 g(H_2_)/kWh, while the highest hydrogen production was 99.3 g(H_2_)/h.

## Introduction

The landscape of global energy is currently dominated by fossil fuels, contributing to approximately 80% of the world’s energy^1,2^. For decades, fossil fuels such as crude oil, natural gas, and coal have been the primary means of energy production. However, the combustion of these fossil fuels comes at a significant environmental cost. The combustion process releases several by-products that have detrimental effects on both the natural environment and human health. The most concerning by-products include carbon dioxide (CO_2_), carbon monoxide (CO), sulphur dioxide (SO_2_), nitrogen oxides (NO_x_), particulate matter and heavy metals.

The natural concentration of carbon dioxide in the Earth’s atmosphere is approximately 0.04%^3,4^. However, due to rapid industrialisation, elevated levels of CO_2_ have been observed, contributing to global climate change. Carbon monoxide is a toxic gas that is both colourless and odourless^5,6^. Exposure to this gas can lead to tissue hypoxia, which poses severe health risks. Sulphur dioxide^6^ is toxic to both animals and humans and contributes to the formation of acid rain and urban smog. Nitrogen oxides^6^, mainly nitrogen dioxide, can cause respiratory issues and contribute to air pollution. Particulate matter^6^, which is composed of fine-grained dust, can pose a risk to respiratory health, causing lung diseases and even cancer. Additionally, heavy metals like lead, radium, and thorium, can also pose serious health risks when released into the atmosphere^7^. The environmental impact of these by-products has led to a global need to transition away from fossil fuel based economies, particularly in the energy sector. The depletion of fossil fuel reserves, coupled with the oil crisis in the 1970s^8^, highlighted the importance of finding new, sustainable, and environmentally friendly energy sources.

In response to the need for future energy sources, three main options are proposed: renewable energy, nuclear power, and clean coal technologies. The ‘hydrogen economy’ can complement each of these options by serving hydrogen as an energy carrier, while fuel cells convert the stored energy in hydrogen molecules into electricity^9–13^. This concept has garnered attention especially in the automotive industry, where hydrogen fuel cells are considered a viable alternative for traditional combustion engines.

The transition from fossil fuels to the hydrogen economy is motivated by: energy source diversification, environmental protection, and price stability. Hydrogen occurs in abundance in many of the earth’s compounds, offering many opportunities for its production, depending on the local sources available. The most significant current issue related to hydrogen production is the reduction of its production cost. The U.S. Department of Energy (DOE)^13,14^ conducted a comprehensive assessment of hydrogen policy, aiming for economically viable technologies with minimal greenhouse gas emissions. To analyse the future development of the hydrogen economy, it is convenient to divide the hydrogen production system into two categories^13,14^:

- the centralised hydrogen production system, i.e. large scale hydrogen production technologies;
- the decentralised (distributed) hydrogen production system, i.e. small and medium scale hydrogen production technologies.

According to the DOE, the accepted industrial cost of hydrogen production is 2 USD/kg(H_2_), equivalent to 60 g(H_2_)/kWh, the so-called target for 2030. The centralised hydrogen production system already meets this criterion. However, this criterion is fulfilled by steam reforming of natural gas, which involves releasing CO_2_ into the atmosphere and is dependent on fossil fuels. In addition, the centralised system requires a suitable infrastructure to deliver the hydrogen to the end user. An alternative solution is to produce hydrogen close to the user. Unfortunately, the methods used in centralised hydrogen production are not easily scalable down due to the use of catalysts and high temperatures. Reducing the production scale results in an increase in the cost of the obtained hydrogen. Therefore, there is a need for an efficient but inexpensive method of hydrogen production on a small or medium scale, close to the end-user. The distributed hydrogen production system avoids costs of hydrogen storage and transport. It is not intended to replace the centralized hydrogen production system, but instead, it is expected to be a significant complement to it in the future.

Currently, methods of distributed hydrogen production do not meet the DOE’s criterion of 60 g(H_2_)/kWh. Moreover, many methods of hydrogen production in the distributed system have the drawback of releasing large amounts of CO_2_ into the atmosphere. The CO_2_ is released into the atmosphere directly during the hydrogen production process and indirectly during the generation of electrical energy necessary for the hydrogen production process. This situation creates a paradox where hydrogen as an energy carrier does not contribute to CO_2_ emissions into the atmosphere, but the processes leading to its production do.

One of the potential methods for decentralised hydrogen production is the plasma method^15–20^. In this method, the plasma generated by electrical discharge converts hydrogen carriers (substances containing hydrogen atoms) into hydrogen. The catalytic properties of plasma result from the presence of high-energy electrons in the plasma, which induce the formation of chemically active particles and radicals^21^. The DOE scenario does not foresee a significant role for plasma technologies in the global hydrogen economy. However, the costs of hydrogen obtained through currently proposed methods for distributed hydrogen production are insufficient to be implemented in the industry. To meet the demand for cost-effective small-scale hydrogen production methods, it is essential to explore new, efficient, and primarily economical approaches. Accordingly, researchers are investigating the use of microwave plasma as a means of generating hydrogen.

The main objective of this work is to gather experimental results regarding the effectiveness of microwave plasma as a method for hydrogen production from conversion of ethanol. Ethanol is an attractive source for hydrogen production due to its high hydrogen atom content, low cost, and wide availability, derived from renewable sources. Its utilisation not only supports sustainable energy practices but also contributes to the development of green technologies. A unique aspect of this work is the exploration of hydrogen production by steam reforming process using plasma generated at the rarely encountered microwave frequency of 915 MHz. The use of 915 MHz microwaves for plasma generation is a relatively less common technique compared to the better known 2.45 GHz frequency. The main advantages of using 915 MHz compared to 2.45 GHz microwaves lie in its deeper penetration depth in the plasma and higher power capabilities. The advantages results in more efficient heating and processing of plasmo-chemical reactions, which is advantageous for applications requiring uniform energy distribution throughout the plasma volume. Additionally, the larger size of 915 MHz equipment allows for higher power capabilities, making it suitable for industrial applications requiring higher power levels. Furthermore, our research is conducted under atmospheric pressure conditions, which makes the presented method more applicable to industrial processes.

The microwave plasma source (MPS) is a device for generating low-temperature microwave plasma at atmospheric pressure^21^. The experimental investigations began with the maximization of the energy efficiency of the MPS. The process of maximization involved determining the position of a movable plunger that ensures the most efficient transfer of microwave energy from the microwave source to the generated plasma in the MPS. This process was supported by computer simulations of the distribution of the electric field module |***E***| within the MPS. Next, tests were conducted to investigate the microwave energy efficiency of hydrogen production from ethanol vapour for the determined position of the movable plunger. The aim was to evaluate the effect of different working conditions of the MPS (include: the absorbed microwave power *P*_A_, the carrier gas flow rate and the amount of ethanol vapour introduced) on the efficiency of hydrogen production.

## Experimental setup

**Figure 1:**
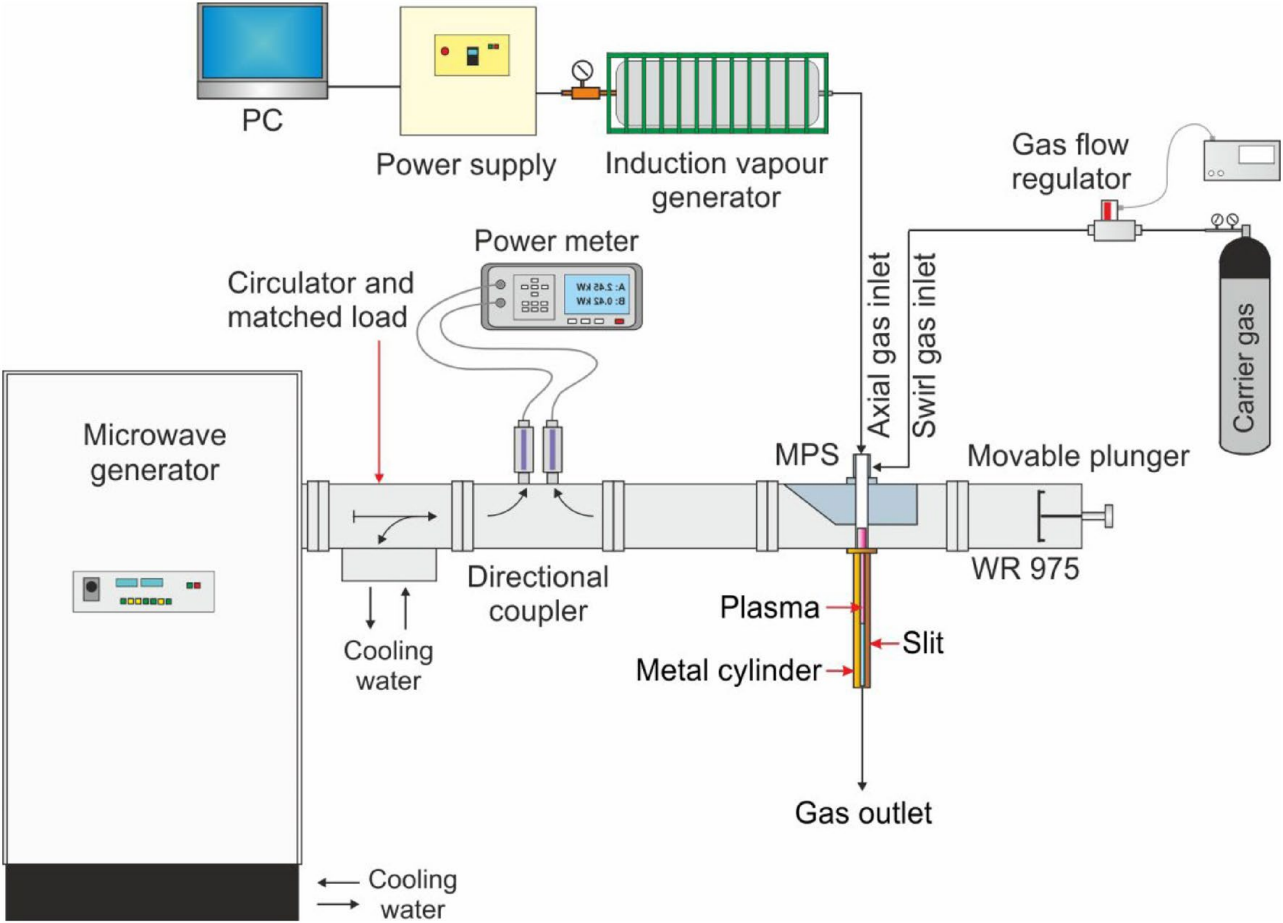
Diagram of the experimental setup.

**Figure 2:**
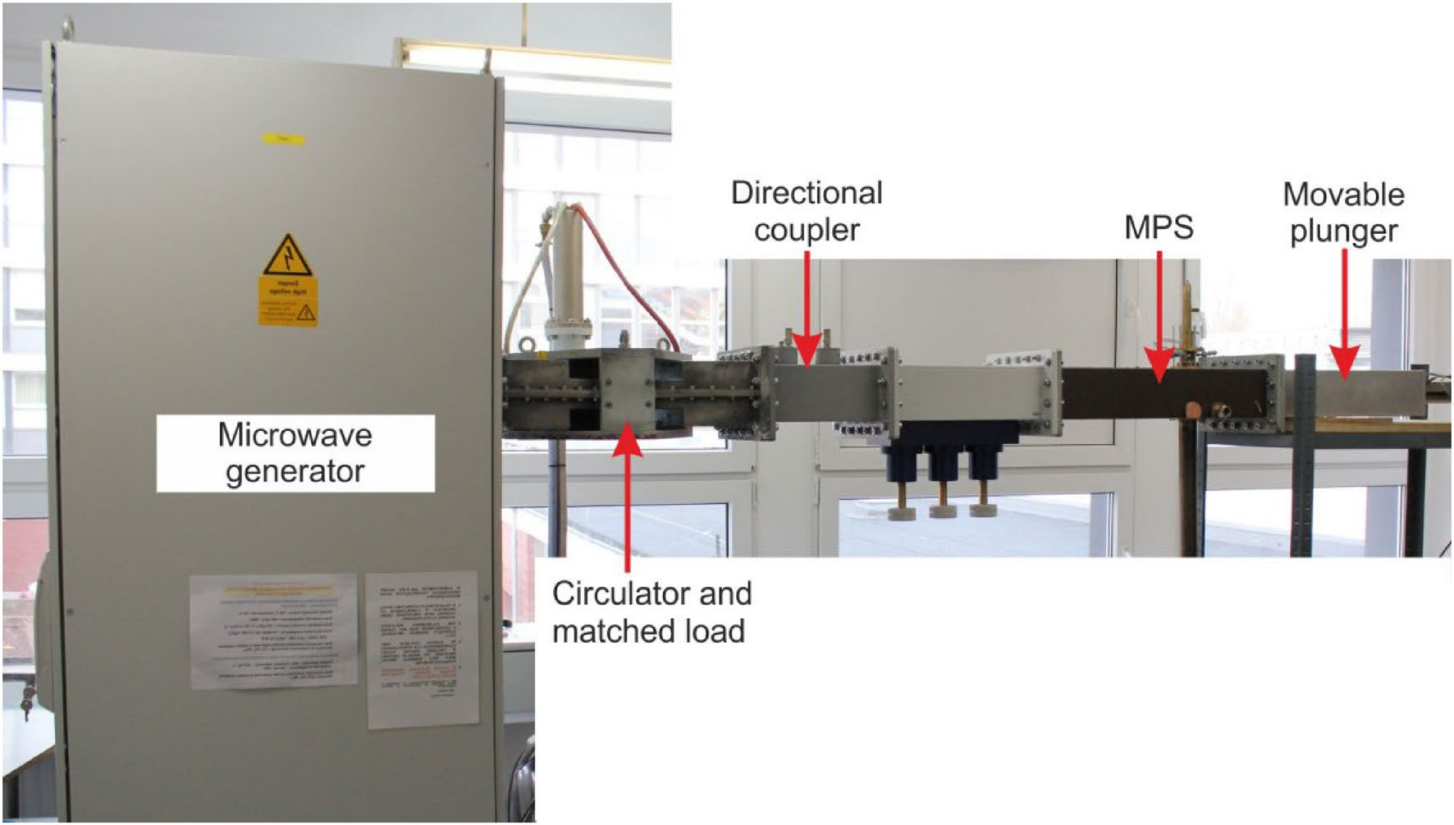
Photo of the experimental setup.

Diagram of the experimental setup is presented in Fig. 1. The setup consists of a microwave generator, a waveguide for microwave transmission, the MPS, and an installation for supplying the working gas (carrier gas + hydrogen carrier in the form of vapours) to the plasma source^22^. A photograph of the setup is shown in Fig. 2.

The microwave generator comprises the following components: a high-voltage power supply, a control panel, and a magnetron. It enables the generation of 915 MHz microwave frequency within a power range of up to 20 kW. The waveguide for microwave transmission is constructed using standard waveguide WR 975 elements with internal dimensions of *a* = 247.7 mm and *b* = 123.9 mm (width × height). The waveguide starts with the magnetron, followed by a circulator with a matched load, a directional coupler connected to a digital dual-channel microwave power meter, the MPS, and the movable plunger. The microwave generator and the circulator with matched load are connected by a common water cooling system. The movable plunger is element of the waveguide that enable minimisation of the reflected microwave power *P*_R_.

**Figure 3:**
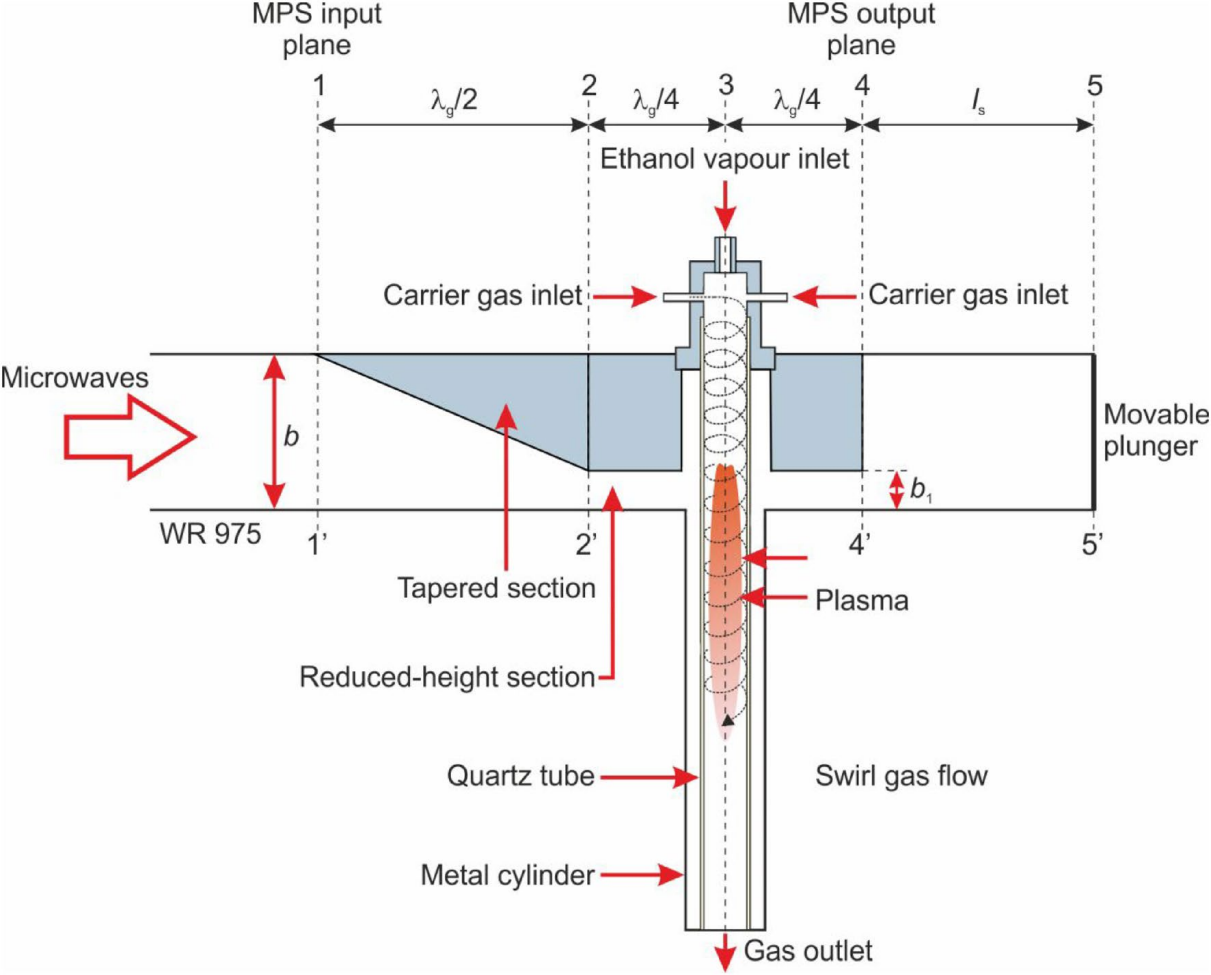
Schematic of the MPS.

In this work, a metal-cylinder-based nozzleless MPS was employed to investigate hydrogen production from ethanol^21,22^. The MPS is based on the standard waveguide WR 975 with a length of λ_g_ (where λ_g_ represents the wavelength of a microwave frequency of 915 MHz in the standard waveguide WR 975, λ_g_ = 437 mm), featuring two additional metal cylinders on the wider side, as illustrated in Fig. 3. The MPS has a tapered section on the magnetron side. Starting from A–A′ plane, this section causes a linear change in the internal height of the waveguide from *b* to *b*_1_ = 31 mm over a length equivalent to λ_g_/2. The height difference, *b*–*b*_1_, matches the height of the subsequently introduced flat middle section, which also has a length of λ_g_/2. In the centre of the middle section, the metal cylinders are introduced containing a quartz tube in which the microwave plasma is generated in the form of flame. The flat middle section creates a reduced-height section inside the MPS which increases the intensity of the microwaves in the area where the plasma flame is generated. A carrier gas, which serves as the medium for initiating and sustaining the microwave plasma, is supplied to the MPS at atmospheric pressure. In this work nitrogen was used as the carrier gas. The presence of a quartz tube prevents the carrier gas from entering the interior of the waveguide. The carrier gas is introduced into the metal cylinder of the MPS above the waveguide through four inlets arranged tangentially to the circumference of the cross-section of this cylinder. This method of gas introduction creates a swirling flow inside the quartz tube, which provides protection against overheating and ensures stabilisation of the generated plasma^21,22^.

The ATW-06/500 inductive vapour generator (manufactured by ALGA) is capable of producing vapours of the liquid hydrogen carrier at temperatures up to 400 °C. The vapours are then supplied axially to the MPS, as shown in Fig. 3. The vapours are introduced into the plasma source through an inlet located in the axis of the metal cylinder above the MPS waveguide—the axial introducing hydrogen carrier vapour.

**Figure 4:**
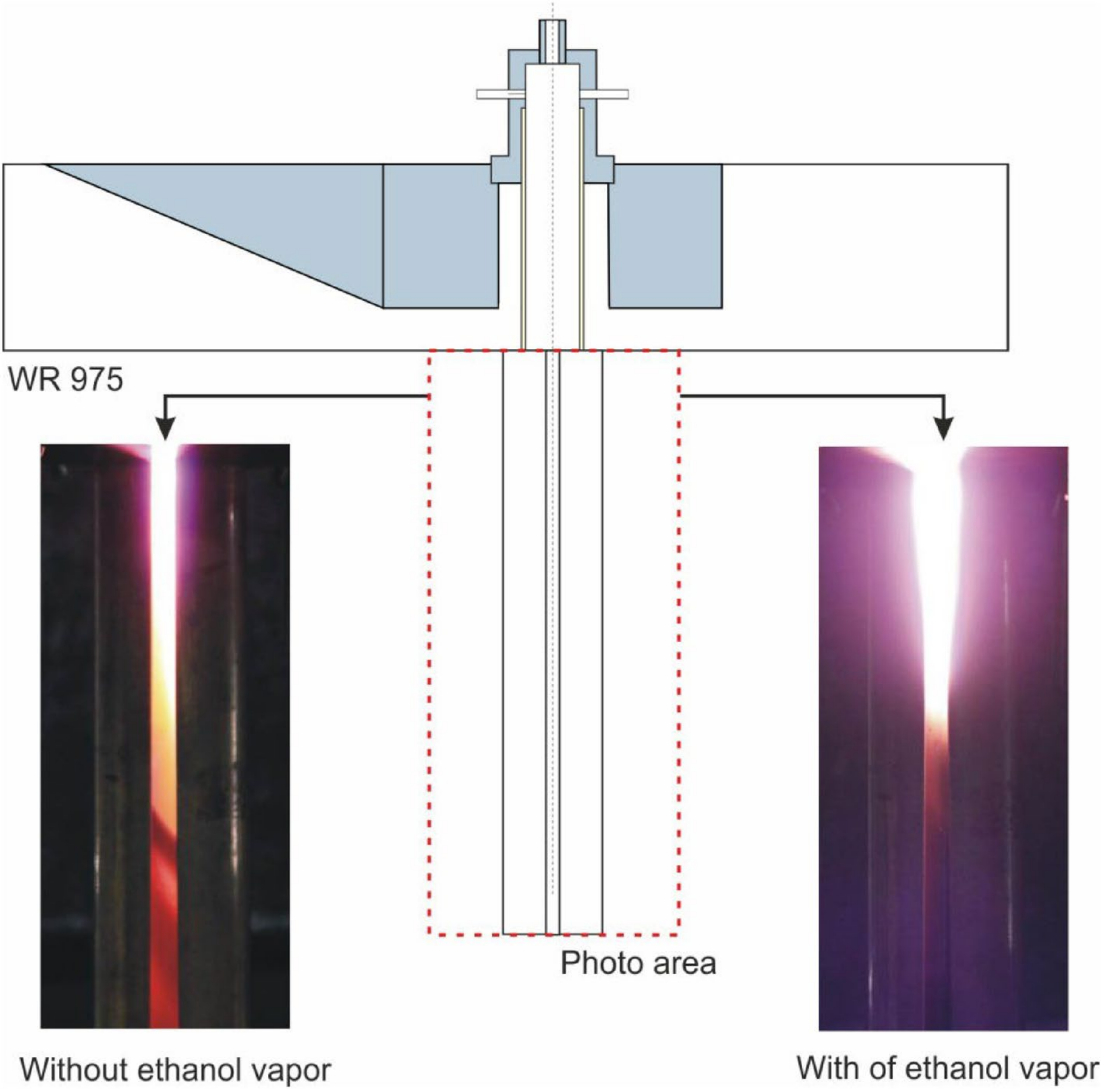
Photos of the microwave plasma generated in nitrogen without and with ethanol vapour addition, nitrogen volumetric flow rate *Q*_N2_ = 2700 NL/h, ethanol vapour flow rate of 0.8 kg/h.

Figure 4 shows photos of plasma flames generated in nitrogen with and without the addition of ethanol vapour observed through the slit of the MPS. The images were taken with the volumetric flow rate of nitrogen *Q*_N2_ = 2700 NL/h and the power *P*_I_ = 5 kW. Ethanol vapour was added to the MPS at a rate of 0.8 kg/h and a temperature of 250 °C.

As illustrated in Fig. 4, the introduction of ethanol vapour resulted in an increase in the intensity of emitted radiation and a change in plasma colour. During the tests, a small amount of carbon deposits was observed on the inner wall of the quartz tube. Furthermore, the presence of ethanol vapour in the microwave plasma resulted in an increase in the observed length of the plasma flame. The experimental work with ethanol vapour in the microwave plasma allowed for the observation of general trends regarding the length of the generated plasma flame. It was found that an increase in the absorbed microwave power *P*_A_ or a decrease in the volumetric flow rate of the carrier gas resulted in an increase in the length of the generated plasma flame.

## Maximization the energy efficiency of the MPS

The cost of obtaining the discharge is a crucial factor in determining the applicability of the microwave plasma in the industry. To reduce the costs, it is essential to maximize the energy efficiency of the MPS. This can be done by identifying the minimum of the electrodynamic characteristics of the MPS. The electrodynamic characteristics refer to the relationship between the ratio *P*_R_/*P*_I_ (where *P*_I_ is the incident microwave power supplied to the MPS input plane) and the distance *l*_s_ from the movable plunger to the MPS, see Fig. 3. The relationship *P*_R_/*P*_I_ (*l*_s_) serves as a metric of the energy efficiency of the MPS, i.e. quantifying the effectiveness of absorbing microwave power by the generated plasma^21,22^. The absorbed microwave power *P*_A_ by the generated plasma is defined as the difference between the power *P*_I_ and *P*_R_. The minimum value in the relationship *P*_R_/*P*_I_ (*l*_s_) indicates the optimal position of the movable plunger, ensuring the most efficient transfer of microwave energy from the microwave generator to the plasma in the MPS. The optimal position of the movable plunger can be determined through experimental measurements of the relationship *P*_R_/*P*_I_ (*l*_s_).

**Figure 5:**
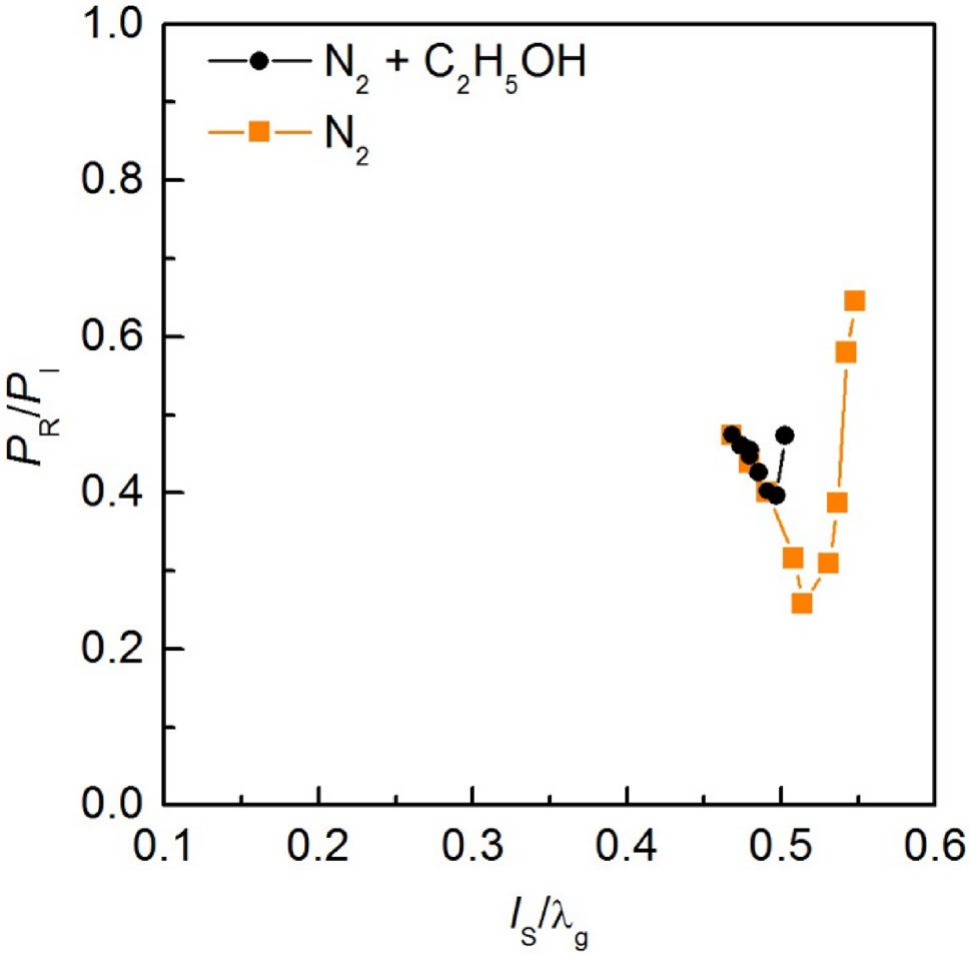
Measured electrodynamic characteristics of the MPS, *P*_I_ = 5 kW, nitrogen volumetric flow rate *Q*_N2_ = 2700 NL/h, ethanol vapour flow rate of 0.8 kg/h.

The relationship *P*_R_/*P*_I_ (*l*_s_) for microwave plasma in nitrogen was measured with and without the addition of the ethanol vapour, Fig. 5. The measurements were carried out under the following conditions: nitrogen with volumetric flow rate *Q*_N2_ = 2700 NL/h, the power *P*_I_ = 5 kW. Ethanol vapour was introduced into the MPS at a rate of 0.8 kg/h and a temperature of 250 °C.

In Fig. 5, the position of the movable plunger was normalised to the wavelength λ_g_. The measured characteristics in nitrogen, with and without the addition of ethanol vapour, indicate the low energy efficiency of the MPS. The minimum of the *P*_R_/*P*_I_ ratio for microwave plasma in nitrogen was greater than 0.25, and the range for stable discharge varied from 0.47 to 0.55. Upon introducing ethanol vapour, the *P*_R_/*P*_I_ ratio increased and was higher than 0.4, also the range of normalised movable plunger positions decrease to range from 0.47 to 0.5. This suggests that the efficiency of microwave energy transfer to the plasma decreases after the introduction of the ethanol vapour and the stability of the generated discharge deteriorates. From a practical standpoint, this means that to sustain plasma in a mixture of nitrogen and ethanol, higher values of the power *P*_I_ need to be supplied to the MPS compared to plasma in pure nitrogen to achieve the same value of the absorbed microwave power *P*_A_. It should also be noted that during the measurement the electrodynamic characteristics of the MPS at lower values of the power *P*_I_ was challenging due to the unstable generation of microwave plasma. This instability prevented the acquisition of the data for lower values of the power *P*_I_ than 5 kW.

### Simulations of the distribution of the electric field module |***E***| in the MPS

Based on the measured electromagnetic characteristics of the MPS, it was concluded that the position *l*_s_/λ_g_ = 0.5 ensures the lowest value of the power *P*_R_ (the minimum of the relationship *P*_R_/*P*_I_ (*l*_s_/λ_g_)). To confirm the maximization the energy efficiency of the MPS at *l*_s_/λ_g_ = 0.5, simulations of the distribution of the electric field module |***E***| inside the MPS were performed. The simulations were carried out to evaluate the effect of the position of the movable plunger and the presence of ethanol vapour on the distribution of the module |***E***| inside the MPS. The simulations were performed based on a two-port equivalent method proposed by Nowakowska et al.^23^, using a model of homogeneous microwave plasma flame^24^, i.e. the concentration of electrons at each point of the plasma column is the same. The transmission properties of such a two-port network can be described using the scattering matrix ***S***. From simulations of the distribution of the electric field modulus |***E***| in the MPS, the elements of the scattering matrix ***S*** can be calculated. The RF module in the COMSL Multiphysics program was used for this purpose^25^.

In a chosen approach, a key factor is assumption of the appropriate shape of the generated plasma flame, an electron concentration *n*_e_ and collision frequency ν, and a value of the relative electrical permittivity *ε*_p_ of the plasma. Based on the observations made during the measurement of the electrodynamic characteristic of the MPS, the generated plasma was assumed to have a column shape with a diameter *d* and height *h*. The adopted values of *d* and *h* for the considered cases of microwave plasma are listed in Table 1.

**Table 1:** The adopted values of the plasma column diameter *d* and height *h.*

Working gas	*d* (cm)	*h* (cm)
Nitrogen	1.2	10
Nitrogen + ethanol vapour	1.2	12

The plasma permittivity *ε*_p_ is assumed to be described by the Lorentz formula^23,24^:

1 $$ \varepsilon_{{\text{p}}} = 1 - n/\left( {1 - {\text{j}}s} \right), $$

where: *n* = *n*_e_/*n*_c_ is the normalised electron concentration relative to the critical concentration *n*_c_, *s* = ν/ω is the normalized electron collision frequency relative to the angular frequency ω = 2π*f*, and a j = (− 1)^1/2^. The *n*_c_ in the plasma is described by the equation^23,24^:

2 $$ n_{{\text{c}}} = \omega^{{2}} \varepsilon_{0} m_{{\text{e}}} /e^{{2}} = {1}.0{4} \times {1}0^{{6}} \;{\text{m}}^{{ - {3}}} , $$

where ε_0_ is the electric permeability of vacuum (ε_0_ = 8.85 × 10^−12^ F/m), *m*_e_ is the mass of an electron (*m*_e_ = 9.1 × 10^−31^ kg), and *e* is the charge of an electron (*e* = 1.6 × 10^−19^ C).

Having established the dimensions of the plasma column and its relative electrical permittivity *ε*_p_, the key parameters that determine the shape of the *P*_R_/*P*_I_ (*l*_s_/λ_g_) relationship are the *n*_e_ and ν, the values of which are currently unknown. The *n*_e_ and ν can be experimentally determined, for example by using optical emission spectroscopy (OES). In cases where experimental conditions do not allow to direct measurement, the values of the *n*_e_ and ν can be estimated by numerically fitting the calculated relationships of the *P*_R_/*P*_I_ (*l*_s_/λ_g_) to the measured electrodynamic characteristics^24^.

**Figure 6:**
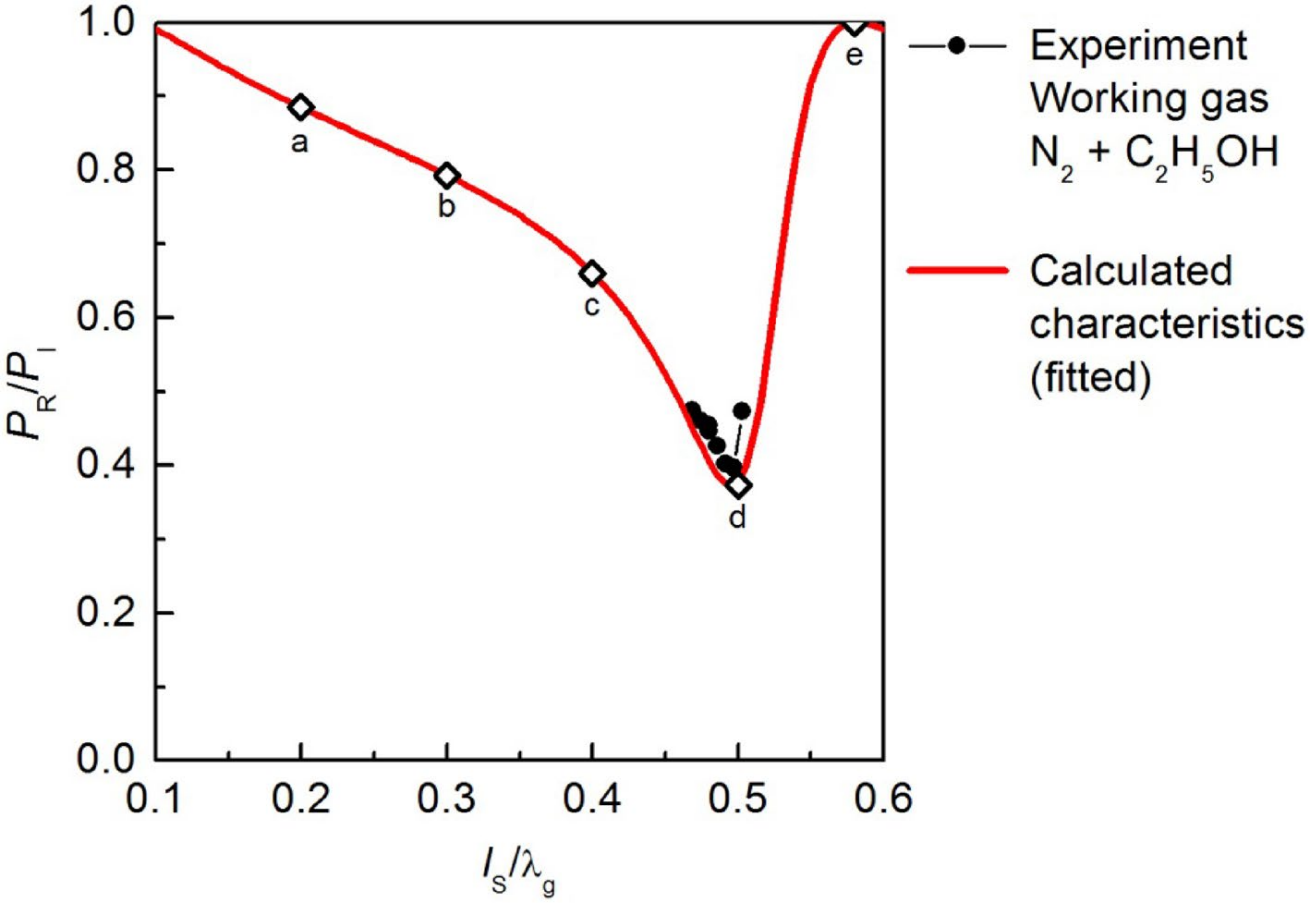
Comparison of the electrodynamic characteristics of the MPS: experimental point and calculated curve; for the marked points: (a), (b), (c), (d) and (e) the distribution of the electric field module |***E***| were simulated.

Following the approach presented in Miotk et al.^24^ and a method of calculating the electrodynamic characteristics developed by Nowakowska et al.^23^, the calculated relationships *P*_R_/*P*_I_ (*l*_s_/λ_g_) were fitted to the measured experimental points by selecting appropriate values for *n* and *s*, as shown in Fig. 6. The least squares method was used as the fitting criterion for the calculated electrodynamic characteristics. The values of the *n*_e_ and ν that gave the best fitting are as follows: in the case of nitrogen, the electron concentration and collision frequency were 2.55 × 10^11^ cm^−3^ and 5.75 × 10^8^ s^−1^, respectively; whereas for nitrogen with ethanol vapour, the values were 2.39 × 10^11^ cm^−3^ and 3.16 × 10^8^ s^−1^. The obtained values of the *n*_e_ and ν showed the following trend: introducing ethanol vapour to the discharge area reduces the value of the electron concentration and collision frequency in the generated plasma.

**Figure 7:**
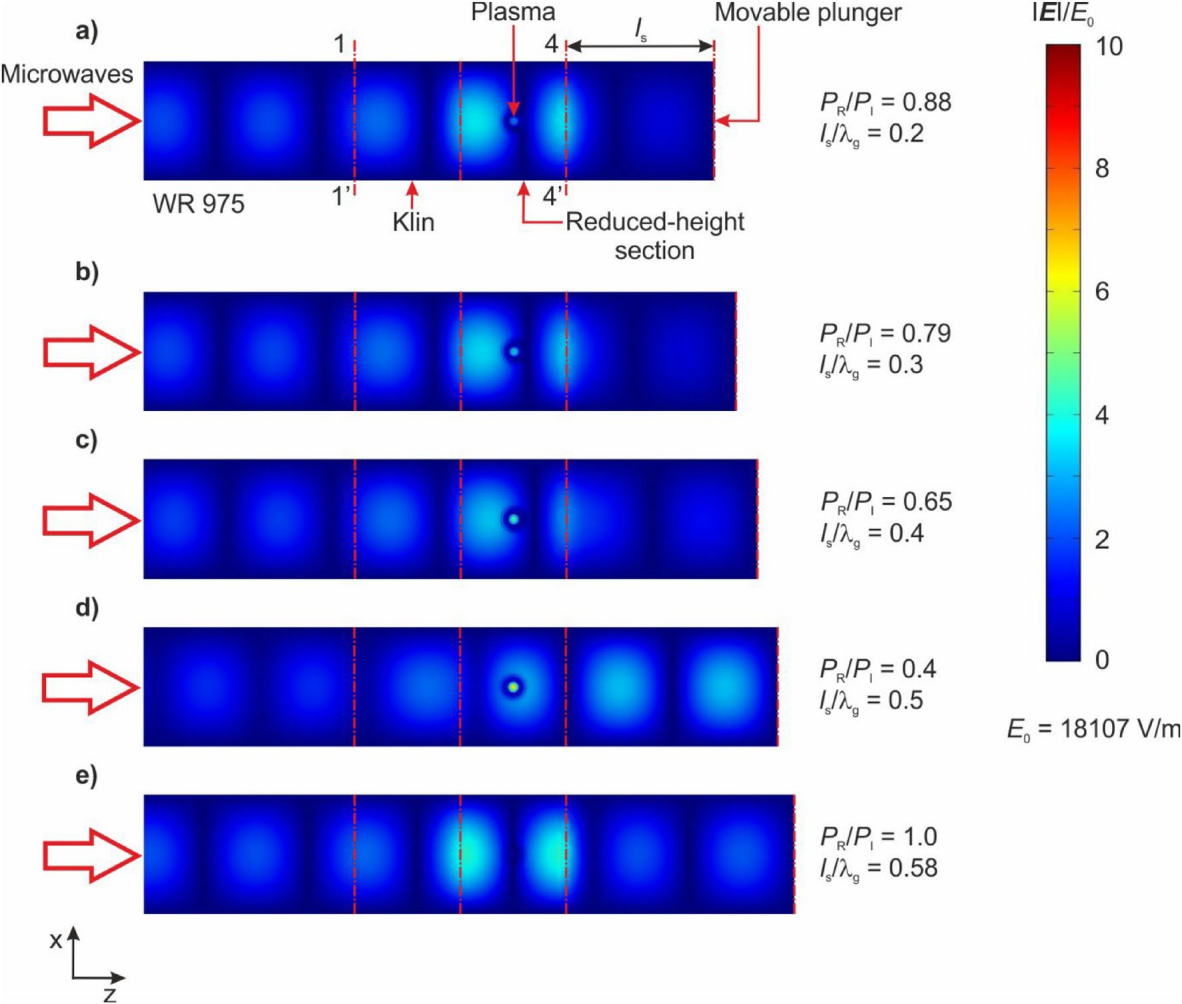
Simulated (top view) normalized electric field module |***E***|/*E*_0_ in the MPS for several movable plunger positions marked as (**a**)–(**e**) on Fig. 5. The simulations refer to the plane located at a height *b*_1_/2 of the reduced height waveguide.

**Figure 8:**
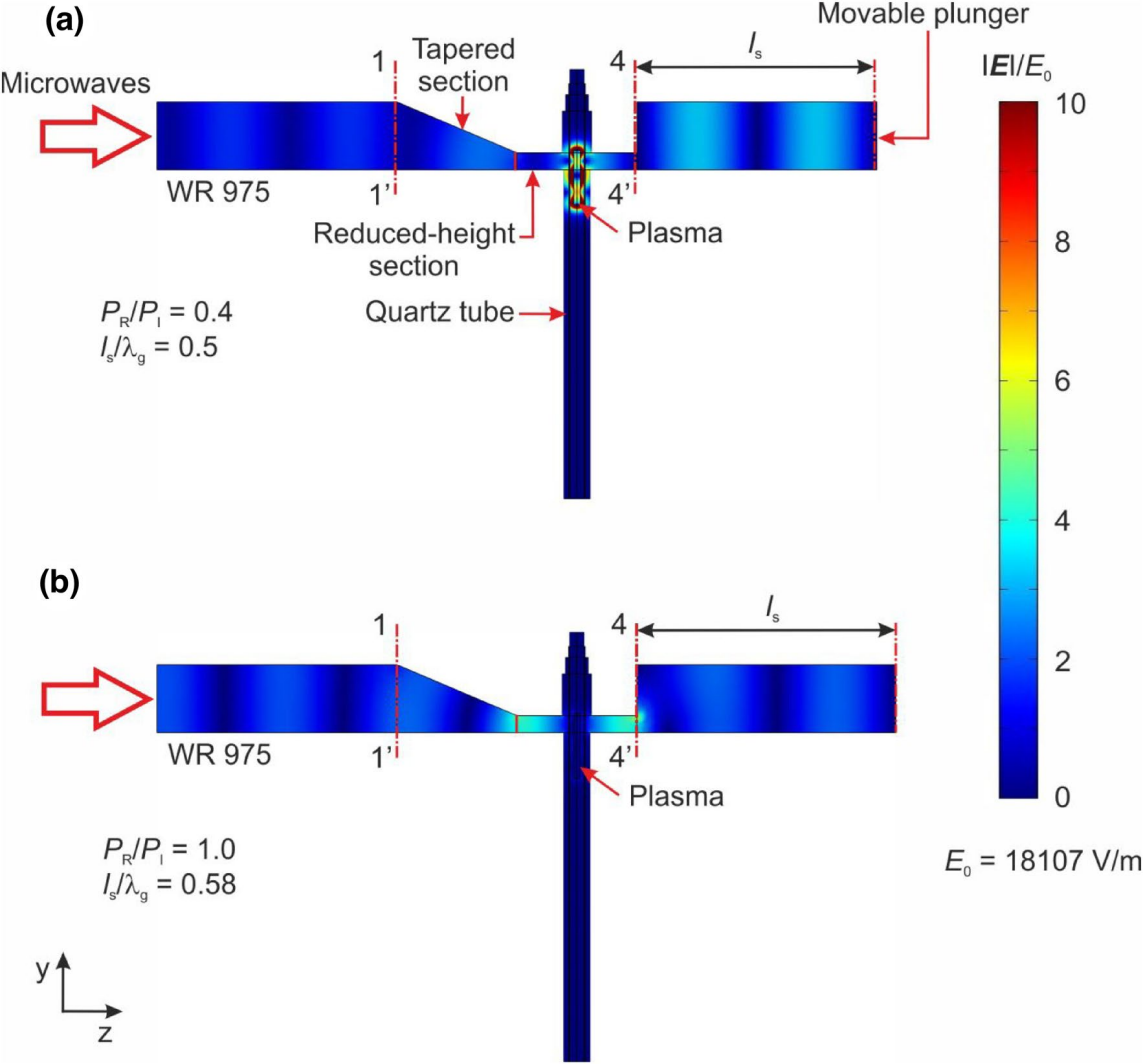
Simulated (cross-section) normalized electric field module |***E***|/*E*_0_ in the MPS for several movable plunger positions marked as (**a**) and (**b**) on Fig. 5. The simulations refer to the plane located at a width *a*/2 of the reduced height waveguide.

By employing the applied plasma model and COMSOL Multiphysics software^25^, simulations of the distribution of the electric field module |***E***| in the MPS were performed. The objective of these simulations was to illustrate the impact of the movable plunger’s position on the distribution of the module |***E***|. The simulations were conducted for normalized positions of the movable plunger, noted as points (*a*), (*b*), (*c*), (*d*) and (*e*) in Fig. 6. The presented simulations were calculated in the case where the working gas was a mixture of nitrogen with added ethanol vapour. The position (*d*) represents the minimum of the electrodynamic characteristics of the MPS. On the other hand, position (e) corresponded to the maximum of the *P*_R_/*P*_I_ (*l*_s_/λ_g_) relationship. The results of the simulations are presented in Figs. 7 and 8. The electric field |***E***| module shown in these figures were normalized to a *E*_0_. The *E*_0_ represents the amplitude of the electric field intensity in a lossless and perfectly matched standard waveguide WR 975 (with inner wall conductivity σ = ∞), where only the ***H***_10_ mode electromagnetic wave propagates. Scaling |***E***|/*E*_0_ enables the comparison of changes in the intensity of the module |***E***| within the MPS relative to the electric field intensity *E*_0_ in the waveguide transmission line. The amplitude *E*_0_ was determined using the relationship^26^:

3 $$ E_{0} = 2\sqrt {\frac{{P_{{\text{I}}} \,Z_{{\text{f}}} }}{a\,b}} , $$

where *Z*_f_ is the impedance of the WR 975 waveguide with internal dimensions of *a* = 247.7 mm and *b* = 123.9 mm. The value of *Z*_f_ can be determined using the following equation:

4 $$ Z_{{\text{f}}} = \eta \left[ {1 - \left( {\frac{{\lambda_{0} }}{2a}} \right)^{2} } \right]^{{ - \frac{1}{2}}} = 502.9\;\Omega , $$

where: *η* ≈ 377 Ω is the impedance of free space, λ_0_ = 327.9 mm is the microwave wavelength at the frequency 915 MHz in free space. Using Eq. 3, for *P*_I_ = 5 kW the amplitude *E*_0_ was equal to 18,107 V/m.

The use of the |***E***|/*E*_0_ scale in Figs. 7 and 8 allowed to observation of a “focusing” effect of the electromagnetic field inside the MPS. This phenomenon is characterized by a significant increase of the |***E***|/*E*_0_ values around the plasma column at the movable plunger position (*l*_s_/λ_g_ = 0.5), which corresponds to the most efficient absorption of microwave power by the generated plasma (minimum *P*_R_/*P*_I_), Figs. 7d and 8d. At this position, the intensity of the module |***E***| is approximately ten times higher than *E*_0_, with |***E***| ≈ 10 × *E*_0_. On the other hand, Figs. 7e and 8e demonstrate simulations when the *P*_R_/*P*_I_ ratio is close to one, indicating that all microwave power delivered to the input plane of the MPS was reflected. In this case the presented distributions show a complete disappearance of the electromagnetic field in the vicinity of the plasma column.

The simulation of the distribution of the module |***E***| in the MPS revealed that for the experimentally determined optimal movable plunger position, the simulated electromagnetic field reaches its peak in the area of microwave plasma generation, ensuring optimal conditions for plasma generation. Investigations into hydrogen production from ethanol vapour were carried out using this established movable plunger position, *l*_s_/λ_g_ = 0.5.

The analysis highlighted the need to improve the MPS in order to increase the transfer of microwave energy from the microwave source to the plasma. Increasing the transfer can be achieved by modifying the construction of the MPS. The modification of the construction of the MPS involves finding internal dimensions that ensure the lowest value of the ratio *P*_R_/*P*_I_ in wide range of the *l*_S_. This can be done experimentally, which is expensive due to the physical search for the most optimal internal dimensions of the device or it can be done by first using simulations of the electric field module |***E***| in the MPS. The simulations enable to prediction of the impact of changes made inside the MPS on the relationships of the *P*_R_/*P*_I_ (*l*_S_/λ_g_). This method is more efficient and cost-effective in terms of time and finances compared to experimental approaches. Measurements of the electrodynamic characteristics and estimation of the *n*_e_ and ν values are the first step in modifying the construction of the MPS using simulations. An example of such modifying can be found in Miotk et al.^27^.

## Experimental results

This section presents the experimental results of hydrogen production from ethanol in microwave plasma. The investigations tested the following working conditions of the MPS: the power *P*_A_, the carrier gas volumetric flow rate, and the amount of the introduced ethanol vapours on the efficiency of hydrogen production. The aim was to achieve maximum efficiency in the hydrogen production, which was characterised by the following parameters:

- production rate (g(H_2_)/h), the amount of hydrogen produced per unit of time;
- percentage of hydrogen in the working gas after plasma treatment:
  $$ [\left( {Q\left( {{\text{H}}_{{2}} } \right)_{{{\text{out}}}} /Q\left( {{\text{gases}}} \right)_{{{\text{out}}}} } \right] \times {1}00\% , $$

where *Q*(H_2_)_out_ is the hydrogen flow rate at the plasma source outlet, and *Q*(gases)_out_ is the total flow rate of working gas at the MPS outlet;

- energy yield (g(H_2_)/kWh), an amount of hydrogen produced using 1 kWh of microwave energy absorbed by the generated plasma;where *Q*(EtOH)_in_ represents the flow rate of the ethanol vapour introduced into the MPS, while *Q*(EtOH)_out_ is the flow rate of the ethanol vapour at the plasma source outlet;
- conversion rate of the ethanol (%): this refers to the proportion of the initial ethanol vapour that undergoes conversion in the plasma:
  $$ \left[ {\left( {Q\left( {{\text{EtOH}}} \right)_{{{\text{in}}}} - Q\left( {{\text{EtOH}}} \right)_{{{\text{out}}}} } \right)/Q\left( {{\text{EtOH}}} \right)_{{{\text{in}}}} } \right] \times {1}00\% , $$
  where *Q*(EtOH)_in_ represents the flow rate of the ethanol vapour introduced into the MPS, while *Q*(EtOH)_out_ is the flow rate of the ethanol vapour at the plasma source outlet;

- selectivity of the ethanol conversion to hydrogen (%): this indicates the proportion of the converted ethanol vapour that has been converted to hydrogen:
  $$ \left[ {Q\left( {{\text{H}}_{{2}} } \right)_{{{\text{out}}}} /Q\left( {{\text{H}}_{{2}} } \right)_{{{\text{in}}}} } \right] \times {1}00\% , $$
  where *Q*(H_2_)_out_ represents the flow rate of the hydrogen at the plasma source outlet, while *Q*(H_2_)_in_ is the flow rate of the hydrogen in the ethanol vapour that introduced into the MPS.

Gas chromatography and IR spectrometry were used to determine the volumetric composition of the working gas after plasma treatment. Each gas sample was analysed at least three times. Thus, the results represent an average value. The precision of the working gas components concentrations was estimated by calculating standard deviation. Furthermore, due to the calibration procedure of the gas chromatography and IR spectrometry used error in gas components concentrations is finally estimated within a range of ± 5%.

This composition provides the necessary information to calculate the hydrogen production efficiency parameters.

The investigation of hydrogen production was carried out using processes of steam reforming of ethanol^27–29^:

- steam reforming—is a process of converting hydrogen carriers into molecular hydrogen in the presence of water vapour, in the case of ethanol, this process may follows as:
  5 $$ {\text{C}}_{{2}} {\text{H}}_{{5}} {\text{OH}} + {\text{3H}}_{{2}} {\text{O}} \to {\text{6H}}_{{2}} + {\text{2CO}}_{{2}} , $$
  6 $$ {\text{C}}_{{2}} {\text{H}}_{{5}} {\text{OH}} + {\text{H}}_{{2}} {\text{O}} \to {\text{4H}}_{{2}} + {\text{2CO}}. $$

The investigations of hydrogen production from ethanol vapour using steam reforming method showed a conversion rate of the ethanol over 99%, regardless of working conditions of the MPS. This means that the ethanol molecules were almost completely decomposed by the microwave plasma generated in our MPS.

**Figure 9:**
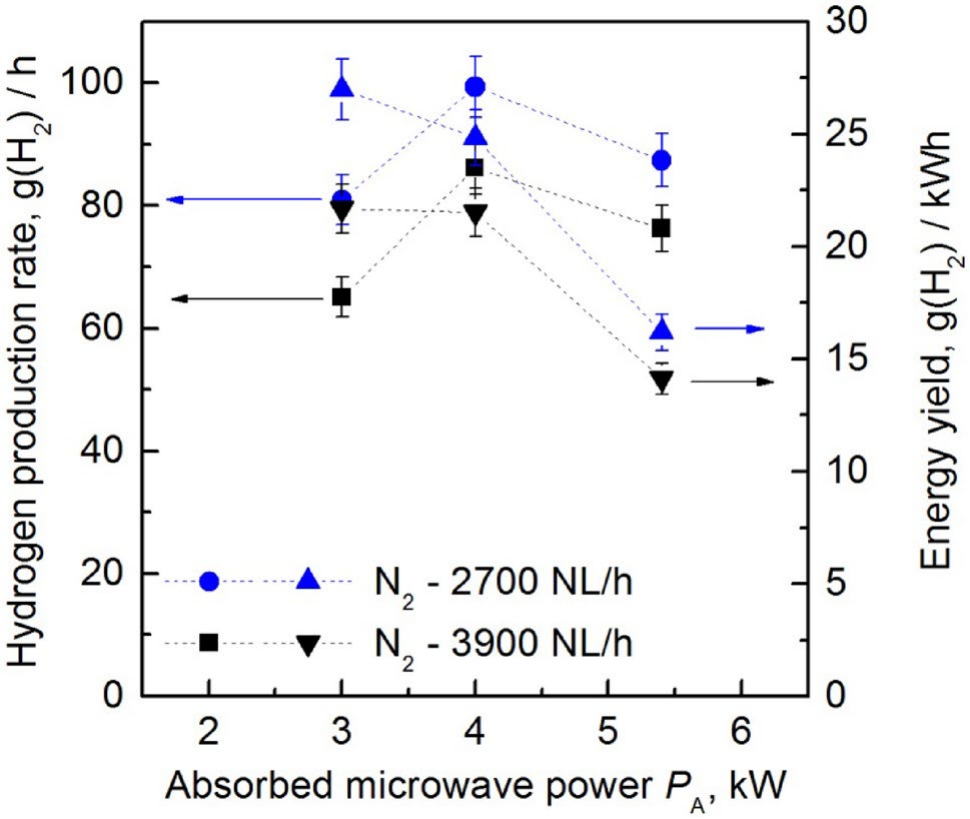
Hydrogen production rate and energy yield as a function of absorbed microwave power *P*_A_ in the process of steam reforming of ethanol vapour. The flow rate of ethanol vapour introduced into the MPS was 0.4 kg/h.

**Figure 10:**
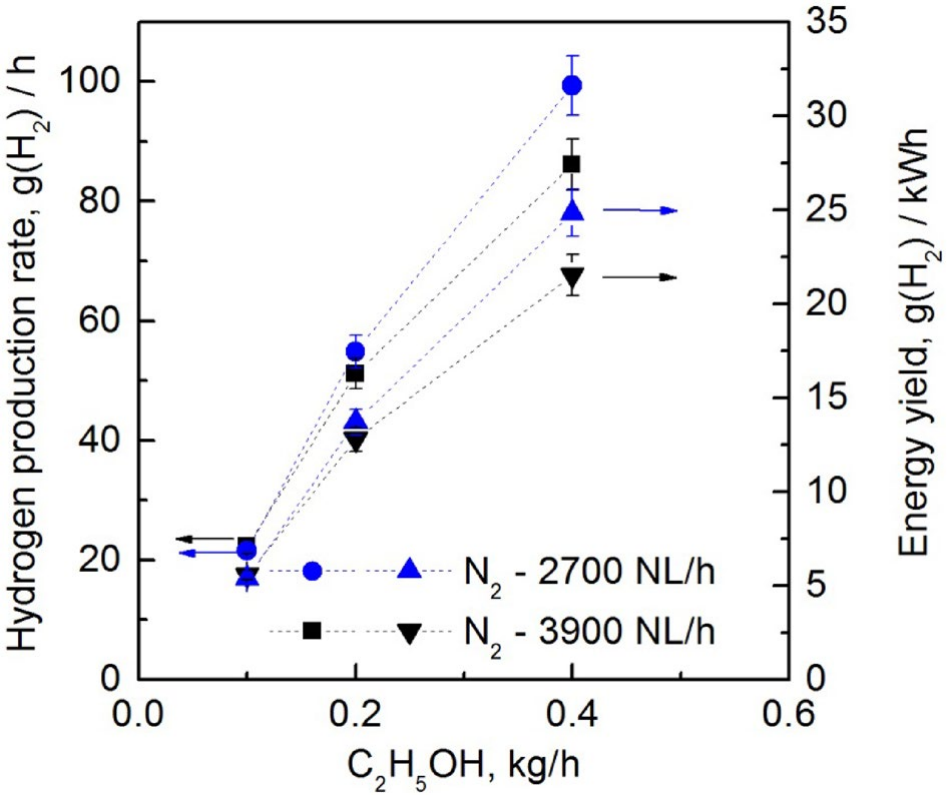
Hydrogen production rate and energy yield as a function of flow rate of ethanol vapour introduced into the MPS in the process of steam reforming. The absorbed microwave power *P*_A_ = 4 kW.

Figures 9 and 10 shows the experimental results of hydrogen production by steam reforming of ethanol. In the tests the ratio of ethanol to water was 1:1 v/v liquid. Then, at a temperature of 250 °C, this mixture was introduced axially into the MPS in the form of vapours. The hydrogen production rate and energy yield were tested as a function of the absorbed microwave power *P*_A_ by the generated plasma for two flow rates of nitrogen (the carrier gas): 2700 NL/h and 3900 NL/h, Fig. 9. Figure 10 shows the relationship between the hydrogen production rate, energy yield, and the amount of ethanol vapour introduced into the MPS. In our experiments, it proved impossible to maintain a stable microwave discharge at the ethanol flow rate above of 0.4 kg/h. This limitation restricts the potential for higher hydrogen production rates. This limitation may be due to an inappropriate ethanol/water ratio, which was 1:1 v/v in the test.

In Fig. 10, the hydrogen production rate and energy yield are strongly dependent on the amount of ethanol vapour introduced. These two important parameters of hydrogen production efficiency increased with the amount of introduced ethanol into the MPS. On the other hand, the increase in the absorbed microwave power *P*_A_ by the generated plasma led to higher hydrogen production rates and a decrease in the energy yield of the obtained hydrogen, as shown in Fig. 9. However, above 4 kW of absorbed microwave power *P*_A_, hydrogen production decreases. This observed decrease in hydrogen production can be attributed to the high microwave power level, which increases the concentration of high-energy electrons. This results in more frequent ionisation and recombination events, altering reaction equilibria and favouring non-hydrogen pathways.

The water vapour added to the discharge, along with ethanol, served as a source of hydrogen in the plasma. As a result, the hydrogen produced in the plasma came from decomposition of the ethanol and water molecules. Therefore, the reported selectivity value refers to the mixture of ethanol and water, and ranges from 60.1% to 100%. The results show that the hydrogen production parameters increase with the amount of ethanol vapour introduced into the MPS. The highest hydrogen production parameters (production rate; energy yield) achieved in the steam reforming of ethanol method were 99.3 g(H_2_)/h and 26.9 g(H_2_)/kWh, as shown in Table 2. In addition, higher values of hydrogen production parameters were achieved for lower volumetric flow rates of nitrogen, as shown in Figs. 9 and 10.

**Table 2:** The highest hydrogen production rate and energy yield achieved in the steam reforming of ethanol.

MPS working conditions	Production rate (g(H_2_)/h)	Energy yield (g(H_2_)/kWh)	Selectivity (%)	Working gas after plasma treatment (% v/v)
N_2_—2700 NL/h C_2_H_5_OH—0.4 kg/h C_2_H_5_OH/H_2_O—1:1 v/v liquid *P*_A_ = 4 kW	**99.3**	24.8	91.9	N_2_—67.3 H_2_—24.1 O_2_—0.6 CO_2_—1.6 CO—7.5 C_2_H_2_—0.1 C_2_H_6_—0.1
N_2_—2700 NL/h C_2_H_5_OH—0.4 kg/h C_2_H_5_OH/H_2_O—1:1 v/v liquid *P*_A_ = 3 kW	81.0	**26.9**	74.9	N_2_—70.8 H_2_—20.4 O_2_—1.3 CO_2_—1.2 CO—6.3 C_2_H_2_—0.1 C_2_H_4_—0.1 C_2_H_6_—0.1

Significant values are in bold.

**Figure 11:**
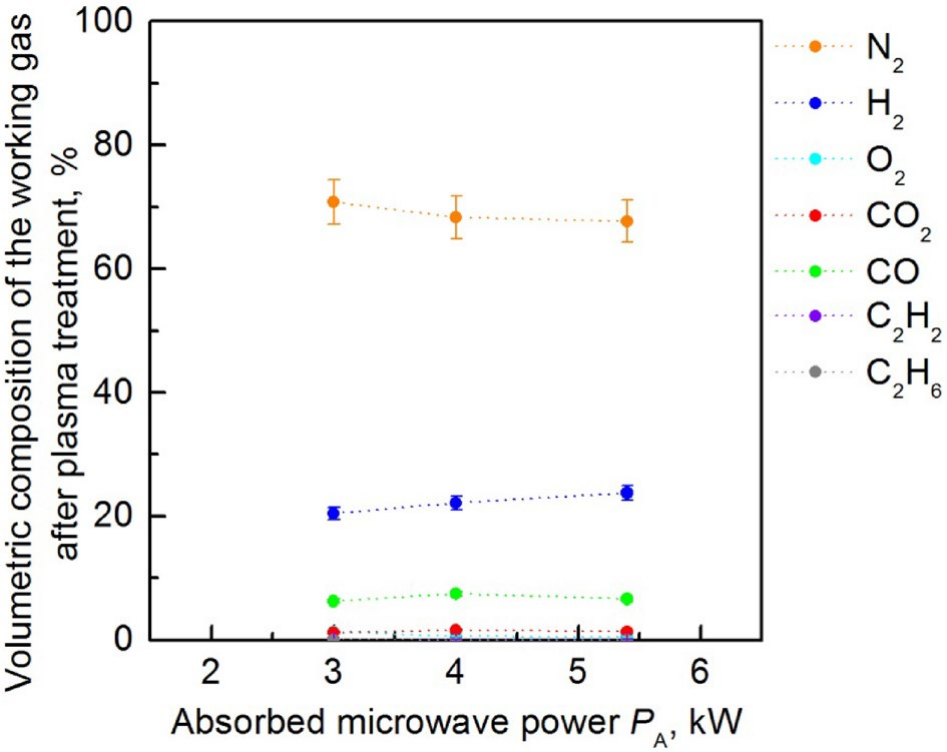
Volumetric composition of the working gas after plasma treatment as a function of absorbed microwave power *P*_A_ in the process of steam reforming of ethanol vapour. The flow rate of ethanol vapour introduced into the MPS was 0.4 kg/h, nitrogen volumetric flow rate *Q*_N2_ = 2700 NL/h.

**Figure 12:**
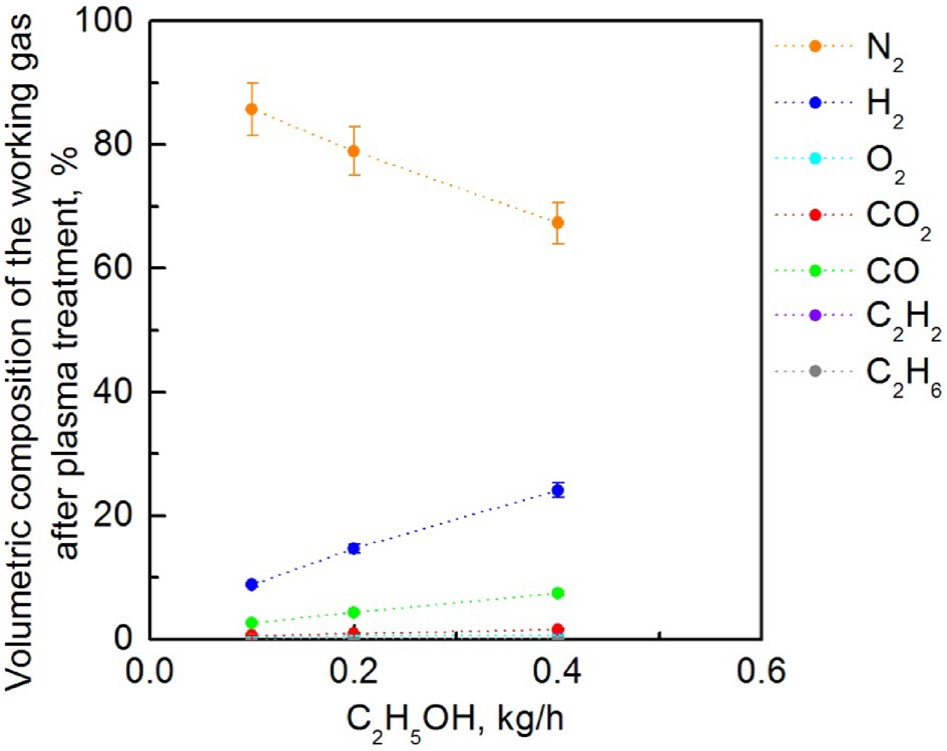
Volumetric composition of the working gas after plasma treatment as a function of flow rate of ethanol vapour introduced into the MPS in the process of steam reforming. The absorbed microwave power *P*_A_ = 4 kW, nitrogen volumetric flow rate *Q*_N2_ = 2700 NL/h.

Figures 11 and 12 show the volumetric composition of the working gas after plasma treatment as a function of the absorbed microwave power *P*_A_ and flow rate of ethanol vapour, respectively. The figures show that the main components of the working gas after plasma rotation are: nitrogen (up to 80% v/v), hydrogen (up to 25% v/v) and carbon monoxide (up to 8% v/v). Other components such as carbon dioxide, oxygen or acetylene (C_2_H_2_) are present in concentrations up to 2% v/v. In the tested range of working conditions of the MPS the amount of hydrogen in the working gas after plasma treatment varied from 6.3 to 25.7% v/v.

Table 3 summarises the impact of the working conditions of the MPS on the efficiency of hydrogen production parameters. The investigations showed that the increase in the absorbed microwave power *P*_A_ by the generated plasma resulted in higher hydrogen production rate, selectivity, and volumetric percentage of hydrogen in the working gas after plasma treatment. This phenomenon is explained as follows: an increase in the absorbed microwave power *P*_A_ lengthens the plasma column, resulting in a longer residence time of the reactants in the discharge region. Furthermore, increasing the amount of ethanol vapour introduced into the MPS resulted in an increase in all parameters characterising the efficiency of hydrogen production except selectivity. Moreover, an increase in the flow rate of nitrogen led to a decrease in all efficiency parameters that characterise hydrogen production efficiency.

**Table 3:** The impact of increasing the tested working conditions of the MPS on the efficiency of hydrogen production parameters.

Tested working conditions	Production rate	Energy yield	Selectivity	H_2_ in the working gas after plasma treatment
Absorbed power *P*_A_	Increase	Decrease	Increase	Increase
Amount of the introduced ethanol vapours	Increase	Increase	Decrease	Increase
Flow rate of nitrogen	Decrease	Decrease	Decrease	Decrease

The efficiency of hydrogen production is significantly influenced by the flow rate of ethanol vapour and the absorbed microwave power *P*_A_. Higher flow rate of ethanol vapour facilitate hydrogen production by increasing the availability of reactants. However, excessive levels can result in plasma saturation and instability. Conversely, higher absorbed microwave power* P*_A_ values boost reaction rates by generating more high-energy electrons, but excessive power can lead to overheating and reduced efficiency. A balance is therefore required: the ethanol vapour concentration should be high enough to ensure an adequate supply of reactants, but not so high as to cause saturation or instability. Concurrently, the absorbed microwave power *P*_A_ must be sufficient to sustain the energetic plasma without causing overheating.

The DOE requires a minimum energy yield of 60 g(H_2_)/kWh for hydrogen production technology to be accepted by the industry, whether in distributed or centralized systems. Table 4 compares the energy yield of hydrogen production from liquid hydrogen carriers using plasma methods. The term ‘catalyst’ in parentheses in the ‘Production method’ column indicates the use of a catalyst in the method, which increases the efficiency of hydrogen production. However, opinions are divided on the potential of catalysts in commercial applications to support hydrogen production using plasma methods^1^. The high cost of the catalyst and its susceptibility to contamination can reduce its effectiveness and contribute to the overall operating costs of the process.

**Table 4:** Comparison of selected plasma methods of hydrogen production from liquid hydrogen carriers.

Production method	Initial composition	Production rate (g(H_2_)/h)	Energy yield (g(H_2_)/kWh)	References
Conventional steam reforming of methane (catalyst)	CH_4_ + H_2_O + air		60	Randolph, U. S. DOE^13^
Water electrolysis	H_2_O		20	Randolph, U. S. DOE^13^
Corona discharges	CH_3_OH + H_2_O		35.71	Liu et al.^30^
Glow discharge	CH_3_OH		75.66	Yan et al.^31^
DC discharge	CH_3_OH + H_2_O + N_2_		24.14	Meiyazhagan et al.^32^
Gliding arc	H_2_O + Ar	0.004	13	Burlica et al.^33^
Gliding arc	CH_3_OH + N_2_		29.9	Zhang et al.^34^
Gliding arc	CH_3_OH + N_2_		32	Zhang et al.^35^
Gliding arc	C_3_H_7_OH + H_2_O + N_2_	1.7	3.3	Pang et al.^36^
Spark discharges	Kerosene		4.2	Malik et al.^37^
Spark discharges	C_2_H_5_OH	1.5	52.8	Xin et al.^38^
Spark discharges	CH_3_OH + H_2_O	1.2	32.4	Ulejczyk et al.^39^
Spark discharges	C_2_H_5_OH + H_2_O	1.4	54	Ulejczyk et al.^15^
Microwave (2.45 GHz) plasma torch	C_2_H_5_OH	0.61	0.17	Rincon et al.^20^
Microwave (2.45 GHz) plasma torch	C_2_H_5_OH + N_2_	60.6	14.8	Hrycak et al.^40^
Microwave (2.45 GHz) plasma torch	C_2_H_5_OH + Ar		0.55	Tsyganov et al.^41^
Microwave (2.45 GHz) discharge in liquid	C_2_H_5_OH	0.07	48.32	Zhu et al.^42^
Microwave (915 MHz) plasma torch	C_2_H_5_OH + N_2_	95.7	22.2	Miotk et al.^22^
Microwave (2.45 GHz) discharge in liquid	C_2_H_5_OH CH_3_OH		12 11.6	Wang et al.^43^
Nozzle-type microwave (2.45 GHz) plasma	C_2_H_5_OH	72.5	48.32	Sun et al.^44^
Microwave (2.45 GHz) plasma torch	C_2_H_5_OH + N_2_	86.7	17.4	Czylkowski et al.^45^
Microwave (2.45 GHz) plasma torch	C_2_H_5_OH + air		38.4	Guo et al.^46^
Microwave (2.45 GHz) plasma torch	C_2_H_5_OH + N_2_	58.4	20.9	Niu et al.^47^
Microwave (2.45 GHz) discharge in liquid	C_2_H_5_OH + H_2_O	8.6	14.4	Batukaev et al.^48^
Microwave (915 MHz) plasma torch	C_2_H_5_OH + H_2_O	99.3	26.9	Present work

The Table 4 shows that literature analysis has mainly focused on plasma conversion of liquid substances for hydrogen production from ethanol and methanol. The plasma technologies are not listed in the DOE report, although some are currently approaching the 60 g(H_2_)/kWh requirement. These are glow discharge, spark discharges, and microwave discharges. The methods developed by Yan et al.^31^, Xin et al.^38^, Ulejczyk et al.^15^, Zhu et al.^42^ and Sun et al.^44^ are the closest to meeting the DOE requirements.

The energy yield obtained in this work is below the DOE criterion of 60 g(H_2_)/kWh. It is important to note that the industry is constantly evolving and changing, and the DOE recommendation is not the only factor that determines the acceptance of hydrogen production methods. With the evolution of the industry, additional criteria have emerged, including resource extraction, production, processing, transportation, utilization, retail sales, and waste disposal. These factors can significantly impact the final implementation of a method. Therefore, the energy efficiency of hydrogen production for different technologies is often not the sole parameter determining the competitiveness of a method, and other criteria must be considered. A method with lower hydrogen production efficiency parameters may still be competitive and worth implementing when other criteria outweigh it.

## Summary

Experimental measurement of the electrodynamic characteristics and simulations of the |***E***| field distribution enabled the determination of the movable plunger position that provided the most efficient energy transfer to the generated plasma in the MPS. The position was *l*_s_/λ_g_ = 0.5. The adopted microwave plasma model enabled the estimation of electron concentration *n*_e_ and collision frequency ν in the generated plasma. The conducted studies indicated that the introduction ethanol vapour into the discharge arena led to a decrease in *n*_e_ and ν values. Furthermore, analysing the electrodynamic characteristics indicated the need to modify the construction of the MPS. The aim of these modifications is to improve the efficiency of the transfer of microwave energy from the microwave source to the plasma and to expand the range for stable discharge in the MPS.

The main aim of the presented investigations was to experimentally test the impact of the selected working conditions of the MPS on the efficiency of hydrogen production from steam reforming of ethanol. The highest energy yield of producing hydrogen achieved the rate of 26.9 g(H_2_)/kWh, while the highest hydrogen production rate was 99.3 g(H_2_)/h. It should be noted that the process of steam reforming of ethanol could only be achieved within a limited range of ethanol vapours introduced into the MPS. Stable microwave discharge could not be sustained above 0.4 kg/h, limiting the potential for higher hydrogen production rates. This limitation may result from an improper ratio of ethanol to water, which was tested at 1:1 v/v. Water vapour is a strong microwave absorber and is likely to absorb a significant proportion of the microwave energy transferred to the MPS, leading to instability in the microwave discharge produced. It is worth noting that the theoretical limit of energy yield for hydrogen production in the steam reforming of ethanol vapour is 249 g(H_2_)/kWh, Eq. (5). With a higher amount of ethanol vapour introduced, the steam reforming process is expected to be more efficient than in the present work, which will be the aim of our next investigations.

## Data Availability

The datasets used and/or analysed during the current study available from the corresponding author on reasonable request.
